# The Effects of Combined Physical Exercise on Serum Redox Biomarkers and Leukocyte DNA Damage of Obese Women

**DOI:** 10.1155/2021/6638420

**Published:** 2021-04-01

**Authors:** Carla Nascimento, Milena Simões Peixoto, Luiz Fernando Fonte Boa, Caroline Coelho de Faria, Tulio Senna Fonseca Costa, Leonardo Matta, Andrea Claudia Freitas Ferreira, Rodrigo Soares Fortunato

**Affiliations:** ^1^Instituto de Biofísica Carlos Chagas Filho, Universidade Federal do Rio de Janeiro, Rio de Janeiro, Brazil; ^2^NUMPEX, Campus Duque de Caxias, Universidade Federal do Rio de Janeiro, Rio de Janeiro, Brazil

## Abstract

Obesity is usually linked to oxidative stress, which can lead to damage to biomolecules. The combination of aerobic and strength exercises seems to induce health benefits in obese individuals, but little is known about the effects of combined physical exercise on redox homeostasis and DNA damage in this population. Thus, the aim of the current study was to determine the effects of 16 weeks of combined physical exercise on biomarkers of oxidative stress and DNA damage in obese women. 17 obese women underwent 16 weeks of a combined physical training program, 3 times per week. Anthropometric and biochemical parameters, serum superoxide dismutase (SOD) and glutathione peroxidase activity, plasma 8-isoprostane levels, and DNA and chromosomal damage were evaluated before and after physical training. Combined physical exercise training decreased body weight (83.2 ± 9.6 vs. 80.2 ± 9.6 kg), body mass index (33.8 ± 3.6 vs. 32.6 ± 3.7 kg·m^−2^), body fat (40.2 ± 2.6 vs. 39.0 ± 3.2%), and waist circumference (99.3 ± 9.4 vs. 94.1 ± 8.8 cm), while the fat-free mass was augmented (59.9 ± 2.9 vs. 60.7 ± 3.1 kg). Moreover, blood glucose reduced (113.5 ± 29.6 vs. 107.3 ± 28.9 mg/dL) along with high-density lipoprotein (54.6 ± 18.1 vs. 59.0 ± 18.8 mg/dL), TSH (2.1 ± 1.1 vs. 2.6 ± 1.2 mIU/mL), and free T4 (0.9 ± 0.1 vs. 1.12 ± 0.2 ng/dL) increase after physical exercise training. Plasma 8-isoprostane levels (17.24 ± 7.9 vs. 29.11 ± 17.44 pg/mL) and DNA damage (34.16 ± 7.1 vs. 45.96 ± 5.8% DNA in tail) were also higher after physical training. No changes were observed in chromosomal damage levels. These results suggest that 16 weeks of combined exercise training 3 times per week is effective in reducing body fat but also increases oxidative stress and DNA damage in obese women.

## 1. Introduction

Obesity is characterized as abnormal or excessive fat accumulation that presents a risk to health. The body mass index (BMI) (the division of a person's weight—in kilograms—by the square height—in meters) is classically used to classify obesity condition, which in this context is stratified into five categories: Grade I (BMI between 30 and 34.9 kg/m^2^), Grade II (BMI between 35.0 and 39.9 kg/m^2^), Grade III (BMI ≥ 40 kg/m^2^), Grade IV (BMI ≥ 50 kg/m^2^), and very severe—Grade V (BMI ≥ 60 kg/m^2^) [1]. Obesity has been recognized as a major underlying factor in the pathogenesis of several diseases such as type 2 diabetes, coronary heart disease, stroke, and cancer [2]. Its prevalence has reached pandemic proportions representing an important health threat, and the costs related to the treatment of this disease achieved extremely elevated levels [3–5].

A variety of studies have demonstrated that obese individuals have higher reactive oxygen species (ROS) levels in several tissues when compared to their eutrophic counterparts [6–8]. Moreover, they seem to have lower antioxidant defense than nonobese individuals [9]. The overproduction of ROS along with the impairment of their detoxification leads to increased ROS availability that can cause oxidative modifications in lipids, proteins, and DNA [10]. The interaction between ROS and DNA is related to a wide range of structural modifications in the DNA molecule, such as oxidation of purines and pyrimidines, apurinic/apyrimidinic (abasic) DNA sites, and single- and double-stranded breaks [11]. Epidemiological studies convincingly show that obese adults have an augmented risk of developing several types of cancer. Besides, studies suggest that the genomic instability generated by oxidative stress in obese individuals is a critical factor in the development of this pathology [12].

There is a large body of evidence demonstrating the beneficial effects of periodized physical exercise on obese people health. For instance, the anti-inflammatory response induced by physical exercise can improve general health, affecting blood vessels, neurons, endocrine system, skeletal muscle, adipose tissue, and immune function [13–15]. Furthermore, moderate chronic physical exercise reduces oxidative stress and molecular damage, both by decreasing ROS production and increasing antioxidant capacity [16]. It has been shown that acute physical exercise promotes a transient prooxidative state that is involved in the activation of multiple redox-sensitive signaling pathways responsible for the cellular adaptation, leading to increased resistance to stress in a chronic perspective [17–19]. However, it is determinant to note that individual characteristics, such as gender, age, and pathological conditions, directly influence the physiological outcome induced by physical exercise. Importantly, obesity is related to reduced overall physical capacity, mainly aerobic capacity, which is a risk factor for pathophysiological changes on different organs and in general metabolism [20]. In this regard, obese individuals require more control over the training prescription, once due to their basal low-grade inflammation and redox imbalance they seem to be more susceptible to exercise-induced oxidative stress [21].

The American College of Sports Medicine proposes the prescription of aerobic, muscle strengthening, and flexibility exercises for eutrophic adults, and the combination of aerobic and strength exercises seems to be efficient to induce health benefits in eutrophic and also in obese individuals [22]. It has been reported that combined exercise (aerobic plus strength) reduces the oxidation of biomolecules and DNA damage and increases antioxidant defense [23–25], but to the best of our knowledge, there are no studies performed exclusively with obese women. Thus, the current study is aimed at determining the effects of 16 weeks of combined physical exercise on markers of oxidative stress and DNA damage in obese women.

## 2. Material and Methods

### 2.1. Data Sharing Statement

The data that support the findings of this study are available from the corresponding author upon reasonable request.

### 2.2. Participants

**Figure 1:**
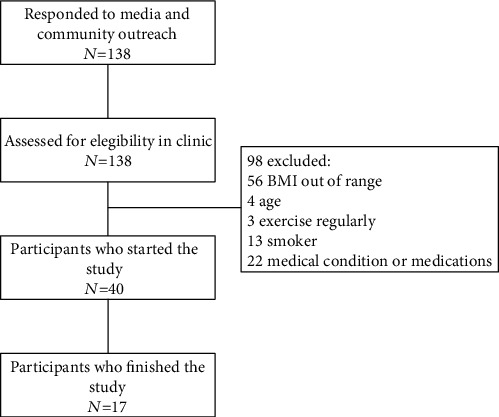
Flow of participants through the trial.

Women aged 32 to 63 years (48 ± 11.7) with a BMI between 30 and 39.99 kg·m^−2^ were recruited. All participants were nonsmoking, not taking hormonal contraceptives, not dieting, and sedentary (defined as exercising ± 40 min per week during the 6 months before baseline testing).  Figure 1 shows the flow of participants through the trial. All participants gave written informed consent. The study was approved by the Clementino Fraga Filho Hospital Ethics Committee for research on humans (approval number: 85529318.1.0000.5257).

### 2.3. Physical Exercise Intervention

**Figure 2:**
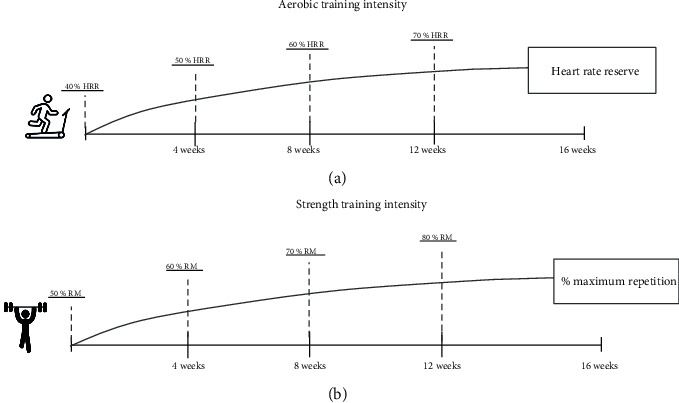
Periodization exercise training. (a) Aerobic training intensity progression based on heart rate reserve (HRR). (b) Strength training intensity progression based on % of maximum repetition.

The subjects followed the exercise training over 16 weeks, with 3 sessions per week, on nonconsecutive days. Each session was divided into 3 parts: aerobic exercise (20 minutes), strength exercise (35 minutes), and stretching exercise (5 minutes). The first treadmill session lasted 15 minutes in weeks 1 to 4 and 20 minutes in weeks 5 to 16. The intensity of aerobic exercise started at 40% of the heart rate reserve (HRR) and increased by 10% every 4 weeks, ending at 16 weeks with 70% of HRR (Figure 2(a)). The strength exercise sequence was hierarchized according to the size of the muscle group, from large to small—bench press, leg press, leg curl, leg extension, *latissimus dorsi*, abdominals, and arm flexion. The intensity of strength training started at 50% of one-repetition maximum (1RM) and increased by 10% every 4 weeks, ending at 16 weeks with 65 to 75% of 1RM (Figure 2(b)). Two sets of each strength exercise were executed in the first 4 weeks, and 3 sets in the following weeks, with 8-12 repetitions and a rest interval of 1 minute between the series.

### 2.4. Anthropometric Measurements

Height (cm) and total body weight (kg) were measured according to the international standards for anthropometric assessment. To evaluate height (cm), a stadiometer (Alturexata) with a scale range of 1 mm was used, and body mass (kg) was measured to the nearest 0.1 kg using a digital scale (Philips, type HF 351/00). Waist circumference (WC) was used taking the umbilicus point as a reference. The evaluation of body composition was performed using the multifrequency electrical bioimpedance apparatus (Biodynamics® model 450).

### 2.5. Blood Sampling

Blood samples were obtained from a forearm vein of each participant in heparinized Vacutainer tubes before and after 16 weeks of exercise training (72 hours after the last exercise session). Blood samples were collected at 8:00 AM after a 12 h fast and immediately centrifuged at 1000 × g for 15 min at ambient temperature to separate serum/plasma from red blood cell pellets. The samples were immediately frozen and stored at -70°C until analysis.

### 2.6. Biochemical and Hormonal Measurements

Blood glucose was analyzed by spectrophotometry (Beckman Coulter). HDL-cholesterol and LDL-cholesterol serum levels were evaluated by colorimetric tests (Beckman Coulter). Glycated hemoglobin was evaluated by high-performance liquid chromatography. Fasting serum thyrotropin (TSH), thyroxine (T4), and free triiodothyronine (T3) levels were measured using specific immunoassays (Immunolite 2000, Siemens). Detection limits were 0.004 *μ*IU/mL, 0.22 ng/dL, and 0.5 pg/mL for TSH, free T4, and total T3, respectively. Serum levels of leptin (range: 62.5–4000 pg/mL) were measured using a commercial enzyme linked-immunosorbent assay (ELISA) kit (Boster Biological Technology Co Ltd., USA; cat. no: EK0437). The intra- and interassay coefficients of variation and sensitivity for leptin were 6.7% and 8%, respectively.

### 2.7. Oxidative Stress Biomarkers

Superoxide dismutase (SOD) activity and glutathione peroxidase (GPX) activity were measured in the serum by colorimetric assay kits (SOD Assay kit, Cayman Chemical Company, catalog number 706002, USA, and GPX Assay kit, Cayman Chemical Company, catalog number 703102, USA), following the manufacturer's instructions. 8-Isoprostane levels were measured in the plasma by a colorimetric assay kit (8-isoprostane assay kit, Cayman Chemical Company, catalog number 516351, USA), following the manufacturer's instructions. The absorbance was read in a microplate reader (Victor X4, PerkinElmer, Norwalk, Conn., USA) at 450 nm for SOD, 340 nm for GPX, and 412 nm for 8-isoprostane. The range of the SOD assay was from 0.005 to 0.050 U SOD/mL, and intra-assay variation was 3.2%. The intra-assay coefficient of variation of the GPX assay was 5.7%. The sensitivity of the 8-isoprostane assay was 3 pg/mL, and intra-assay variation was 7.2%. All samples were measured in the same assay.

### 2.8. Comet Assay

5 *μ*L of whole blood was suspended in 100 *μ*L of 0.5% low melting point agarose in PBS. The cells were then homogenously spread onto two microscope slides precoated with 1.5% agarose, immediately covered with coverslips, and kept at 4°C for 10 min. The solidified slides were immersed in cell lysis buffer (2.5 M NaCl, 100 mM Na 2 EDTA, 10 M Tris, 10% DMSO, 1% Triton X-100, pH 10) at 4°C overnight. The slides were then placed horizontally in an electrophoresis chamber with cold alkaline buffer (300 mM NaOH, 1 mM EDTA, pH > 13) for 20 min, and electrophoresis was performed for 25 min at 25 V and 300 mA. Then, slides were neutralized with three 5 min washes with neutralization buffer (0.4 M Tris, pH 7.5) and fixed with ice-cold 100% ethanol. Finally, the slides were stained with DAPI and imaged with a fluorescence microscope at a magnification of 200x. At least 100 comets per slide were scored, in duplicate, using the OpenComet v1.3.1 software. The results were plotted by the percentage of tail DNA content [26].

### 2.9. Cytokinesis-Block Micronucleus Cytome Assay

The assay was performed following specifications according to the Fenech protocol [27], with some modifications. 400 *μ*L of heparinized whole blood was added to 4.5 mL of DMEM medium plus 100 *μ*L of phytohemagglutinin (Gibco), and the samples were incubated at 37°C and 5% CO_2_. After 44 hours, cytochalasin B at a final concentration of 5 *μ*g/mL was added to the samples and incubated for further 28 hours to block cytokinesis, totalizing 72 hours of incubation. Thereafter, samples were washed with PBS and centrifuged at 1000 rpm for 5 minutes. The supernatant was discarded, and the lymphocytes were collected with ice-cold hypotonic solution (KCl 0.075 M) for red cell lysis. After another centrifugation, the supernatant was discarded and fixed with 5 mL of methanol/acetic acid solution (3 : 1 *v*/*v*), followed by centrifugation at 1500 rpm for 5 minutes for 3 times. Finally, 1 mL of fixative solution was added for the preparation of the slides. The slides were stained with 10% Giemsa solution for optical microscopy analysis. Binuclear cells were scored, as well as micronuclei (MN), nucleoplasmic bridges (NPBs), and nuclear buds (NBUDs), as proposed by Fenech [27]. 3.000 binucleated cells were scored per treatment, and the results were expressed in the number of occurrences of alterations per 1.000 binucleated cells.

### 2.10. Statistical Analysis

All the results are expressed as the mean ± standard deviation of the mean and were analyzed by a paired *T*-test using the GraphPad Prism software (version 5.01, GraphPad Software Inc., San Diego, USA). The minimum level of significance was set at *P* < 0.05.

## 3. Results

Herein, we report results from the 17 women who completed the entire study. The baseline anthropometric characteristics of the participants are listed in Table 1. As observed, at baseline, participants had a BMI mean of 33.8 kg·m^−2^, which was significantly reduced to 32.6 kg·m^−2^ after the physical exercise intervention. Moreover, body fat percentage and waist circumference were also reduced, while fat-free mass increased after 16 weeks of physical exercise.

The physical exercise protocol was found to decrease blood glucose in the subjects, but there was no effect concerning serum levels of glycated hemoglobin, total lipids, and LDL (Table 2). Besides, serum HDL levels were significantly elevated. Regarding hormonal measurements, free T4 and TSH serum levels were significantly increased by exercise training, and no changes were observed for serum-free T3 and leptin levels.

**Figure 3:**
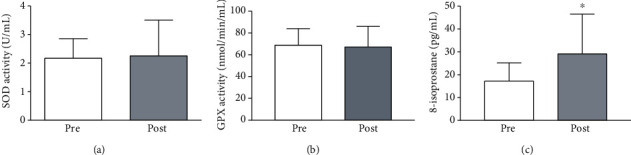
Oxidative stress biomarkers in obese women pre- and post-16 weeks of combined physical exercise. Serum superoxide dismutase activity (a), serum glutathione peroxidase activity (b), and plasma 8-isoprostane levels (c) were measured by spectrophotometry. The results are expressed as the mean ± SD. ^∗^*P* < 0.05.

There was no effect of physical exercise on SOD (Figure 3(a)) and GPX activities (Figure 3(b)). However, 8-isoprostane, a marker of lipid peroxidation, was higher after exercise training (*P* < 0.05) (Figure 3(c)).

**Figure 4:**
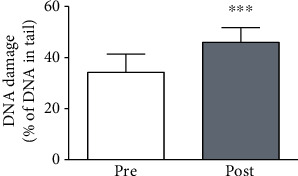
DNA damage in obese women pre- and post-16 weeks of combined physical exercise assessed by the comet assay. The results are expressed as the mean ± SD. ^∗∗∗^*P* < 0.001.

The alkaline comet assay was performed to determine the DNA damage in peripheral blood cells of obese individuals before and after physical exercise training. We noticed that 16 weeks of combined physical exercise training increased DNA damage levels (*P* = 0.0002) in the subjects (Figure 4).

Interestingly, no differences between groups were found for the different parameters evaluated in the micronucleus assay (Table 3).

## 4. Discussion

Women are particularly affected by the obesity epidemic, and their greater prevalence becomes only manifested in adulthood [28]. Recent estimates reveal that the incidence of obesity will reach 18% in men and 21% in women by 2025 [29]. The sex-related differences in obesity have been attributed to nutrition, lifestyle, behavior, and environmental differences between men and women [30, 31]. In the current study, we investigated the effect of 16 weeks of combined physical exercise on redox markers and DNA damage in obese women. Our results demonstrate that the combined exercise intervention was effective in parameters related to body composition, such as a decrease in body weight, BMI, waist circumference, and fat mass, along with elevations of fat-free mass. However, we found an increase in serum 8-isoprostane, a marker of lipid peroxidation, and leukocyte DNA strand breaks.

Our study showed that after 16 weeks of exposure to training, obese women had a reduction in blood glucose, which is a well-established effect of physical exercise once it is capable of enhancing tissue sensitivity to insulin. Besides, alterations in body weight and composition have also been shown to affect tissue sensitivity to insulin [32, 33]. We observed an increase in HDL, which is related to beneficial effects on the metabolism of obese individuals and contributes to reducing chronic low-grade inflammation [34]. Moreover, serum T4 and TSH levels were increased by physical exercise, but not T3 levels. As T3 is the active hormone, thyroid hormone signaling probably was unaltered. Further studies are necessary to explain the observed changes in T4 and TSH. Serum levels of total cholesterol, triglycerides, LDL, leptin, and glycated hemoglobin were not affected by combined exercise, so we postulate that it might necessitate a longer period of intervention to observe an improvement in those parameters.

Herein, we show that 16 weeks of the combined exercise was able to increase lipid peroxidation, without changes in serum GPX and SOD activities on obese women. Regarding the specific literature, few studies have been conducted in obese individuals evaluating the effect of combined exercise training on redox homeostasis. Overweight elderly women who performed moderate-intensity combined aerobic-resistance exercise training 3 times a week for at least 18 months had a decrease in total peroxides and an increase in total antioxidant capacity [24]. Medeiros et al. demonstrated an enhanced lipid peroxidation and a reduction of serum GPX activity in obese individuals submitted to 26 sessions of combined exercise 3 times a week, with no changes in the other two oxidative stress biomarkers, protein carbonyl and sulfhydryl. Interestingly, when the subjects performed the same 26 sessions 5 times a week, no differences were observed in lipid peroxidation between groups, and a decrease of protein carbonyl was found [35]. So, the longitudinal continuity and frequency seem to be a determinant in the modulation of redox homeostasis, once training performed for longer periods and more frequently would be related to better responses.

Moreover, obese individuals are more susceptible to redox homeostasis challenges by having higher ROS levels and decreased antioxidant defense. It has been shown that an acute increase in serum lipids elevates F2-isoprostane levels more markedly in obese than in lean individuals [36]. Physical exercise increases ROS generation acutely, being much more pronounced in obese in comparison to eutrophic subjects due to their basal oxidative redox state [37, 38]. Thus, it is possible that the fine-tuning redox-mediated signaling required to correctly activate the molecular effectors in response to exercise was not reached in our study subjects because of the conditions described above. The lack of effect of physical exercise on the activity of the antioxidant enzymes SOD and GPX corroborates this hypothesis. Furthermore, we take into consideration that our sample is composed exclusively of women, and sex would be a pertinent factor in the physiological response to exercise [39]. Moreover, more studies are necessary to elucidate this question because only 17 individuals were evaluated in our study.

The mechanism by which physical exercise influences DNA stability is not completely elucidated, but the prooxidative state found during/after the exercise session seems to be involved. In our study, utilizing the alkaline comet assay (a method capable of detecting DNA double-strand breaks, single-strand breaks, alkali-labile sites, DNA-DNA/DNA-protein cross-linking, and incomplete excision repair sites at a single cell level), we observed higher levels of leukocyte DNA damage in obese women after 16 weeks of combined exercise [26]. Several investigations reported the occurrence of DNA damage in white blood cells following different types of acute exercise such as high-intensity, short-duration [40–42], moderate-intensity and duration [42], and moderate-intensity endurance [43]. Hartmann et al. showed that healthy men supplemented with vitamin E (1.200 mg daily) for 14 days before a run had their exercise-induced DNA damage completely abrogated in 4 of the 5 volunteers, while the lipid peroxidation increase after exercise did not occur [40]. Another study described a 62% reduction in the DNA damage rate in women taking the antioxidants vitamin C (1.000 mg) and RRR-a-tocopheryl acetate (400 IU) during 6 weeks before an ultramarathon race [44]. Here, we observed an increase in lipid peroxidation after 16 weeks of combined exercise training that may be related to the higher levels of DNA damage, since lipid peroxidation products have been shown to form DNA adducts mediating DNA damage associated with oxidative stress [45]. However, more experiments are needed to prove this hypothesis.

To the best of our knowledge, no studies evaluated the effect of combined exercise training on DNA damage in obese women. We found only one study evaluating the effect of 16 weeks of combined exercise, but the volunteers were healthy men [23]. In this study, combined exercise training led to a decrease in DNA single-strand breaks and FPG-sensitive sites. Once DNA repair was not affected by training, the authors proposed that a reduction of ROS production and/or an increase of antioxidant defense were responsible for decreased oxidative DNA damage, since the exercise intervention reduced lipid peroxidation and elevated total antioxidant capacity [23]. This observation suggests that the lack of adaptation related to redox homeostasis in the present study may be associated with obesity, probably due to the high basal levels of oxidative stress found in these individuals [23]. Besides, a deficient repair of the DNA molecule could also be involved. To gain more insights into the biological significance of the observed effects on DNA by the comet assay, it is necessary to compare these effects with other endpoints of genotoxicity. Thus, we analyzed chromosome breakage and chromosome loss by the micronucleus assay. Despite the results found of augmented DNA damage, no change in micronucleus frequency was detected when comparing pre- and postexercise results. This suggests that DNA lesions are probably repaired and do not result in chromosome damage.

## 5. Conclusions

In summary, we demonstrate that 16 weeks of combined physical exercise performed 3 times per week presented positive effects related to body composition and blood biochemical changes, but it was not sufficient to increase antioxidant defense and resulted in increased lipid peroxidation and DNA damage in obese women, without changes in chromosome breakage. Thus, it is important to note that controlling training variables (volume, intensity, weekly frequency, and duration) should be considered according to individual physiological conditions, especially for obese women.

## Figures and Tables

**Table 1:** Anthropometric characteristics of 17 obese women prior to and following 16 weeks of combined physical exercise.

	Preexercise training	Postexercise training
Height (cm)	156 ± 4.43	N/A
Weight (kg)	83.2 ± 9.6	80.2 ± 9.6^∗∗^
BMI (kg·m^−2^)	33.8 ± 3.6	32.6 ± 3.7^∗∗^
Body fat (%)	40.2 ± 2.6	39.0 ± 3.2^∗^
Fat-free mass (kg)	59.9 ± 2.9	60.7 ± 3.1^∗^
Waist circumference (cm)	99.3 ± 9.4	94.1 ± 8.8^∗∗∗^

^∗^ *P* < 0.05; ^∗∗^*P* < 0.01; ^∗∗∗^*P* < 0.001. Data were expressed as the mean ± SD. BMI: body mass index; LDL: low-density lipoprotein; HDL: high-density lipoprotein; N/A: not applicable.

**Table 2:** Biochemical and hormonal measurements of 17 obese women prior to and following 16 weeks of combined physical exercise.

	Preexercise training	Postexercise training
Glucose (mg/dL)	113.5 ± 29.6	107.3 ± 28.9^∗^
Glycated hemoglobin (%)	5.8 ± 0.9	5.9 ± 1.1
Total lipids (mg/dL)	683.0 ± 228.3	630.1 ± 119.6
HDL (mg/dL)	54.6 ± 18.1	59.0 ± 18.8^∗^
LDL (mg/dL)	140.6 ± 72.2	116.3 ± 28.8
Free T3 (pg/mL)	2.5 ± 0.9	3.2 ± 2.0
Free T4 (ng/dL)	0.9 ± 0.1	1.12 ± 0.2^∗∗∗^
TSH (mIU/mL)	2.1 ± 1.1	2.6 ± 1.2^∗∗^
Leptin (mg/dL)	29.7 ± 4.9	29.8 ± 2.8

^∗^ *P* < 0.05; ^∗∗^*P* < 0.01; ^∗∗∗^*P* < 0.001. Data were expressed as the mean ± SD. LDL: low-density lipoprotein; HDL: high-density lipoprotein; TSH: thyroid-stimulating hormone.

**Table 3:** Number of micronuclei, nuclear buds, and nucleoplasmic bridges in 1000 binucleated blood lymphocytes of 17 obese women prior to and following 16 weeks of physical exercise.

	Preexercise training	Postexercise training
Micronuclei	66.5 ± 45.1	76.5 ± 54.9
Nuclear buds	3.6 ± 7.4	2.8 ± 3.7
Nucleoplasmic bridges	4.0 ± 8.7	3.7 ± 5.6

Data were expressed as the mean ± SD.

## Data Availability

All data used to support the findings of this study are available from the corresponding author upon request.
